# Favorable Effects of a Ketogenic Diet on Physical Function, Perceived Energy, and Food Cravings in Women with Ovarian or Endometrial Cancer: A Randomized, Controlled Trial

**DOI:** 10.3390/nu10091187

**Published:** 2018-08-30

**Authors:** Caroline W. Cohen, Kevin R. Fontaine, Rebecca C. Arend, Taraneh Soleymani, Barbara A. Gower

**Affiliations:** 1 Department of Nutrition Sciences, University of Alabama at Birmingham, Birmingham, AL 35294, USA; tsoleymani@hotmail.com (T.S.); bgower@uab.edu (B.A.G.); 2 Department of Health Behavior, University of Alabama at Birmingham, Birmingham, AL 35294, USA; kfontai1@uab.edu; 3 Department of Obstetrics and Gynecology, University of Alabama at Birmingham, Birmingham, AL 35294, USA; rarend@uabmc.edu

**Keywords:** ketogenic diet, quality of life, physical function, mental function, fatigue, food cravings, ovarian cancer, endometrial cancer

## Abstract

Ketogenic diets (KDs) are gaining attention as a potential adjuvant therapy for cancer, but data are limited for KDs’ effects on quality of life. We hypothesized that the KD would (1) improve mental and physical function, including energy levels, (2) reduce hunger, and (3) diminish sweet and starchy food cravings in women with ovarian or endometrial cancer. Participants were randomized to a KD (70:25:5 energy from fat, protein, and carbohydrate) or the American Cancer Society diet (ACS: high-fiber, lower-fat). Questionnaires were administered at baseline and after 12 weeks on the assigned diet to assess changes in mental and physical health, perceived energy, appetite, and food cravings. We assessed both between-group differences and within-group changes using ANCOVA and paired *t*-tests, respectively. After 12 weeks, there was a significant between-group difference in adjusted physical function scores (*p* < 0.05), and KD participants not receiving chemotherapy reported a significant within-group reduction in fatigue (*p* < 0.05). There were no significant between-group differences in mental function, hunger, or appetite. There was a significant between-group difference in adjusted cravings for starchy foods and fast food fats at 12 weeks (*p* < 0.05 for both), with the KD group demonstrating less frequent cravings than the ACS. In conclusion, in women with ovarian or endometrial cancer, a KD does not negatively affect quality of life and in fact may improve physical function, increase energy, and diminish specific food cravings. This trial was registered at ClinicalTrials.gov as NCT03171506.

## 1. Introduction

Although ketogenic diets (KDs) have been in medical use for almost a century in pharmacoresistant epilepsy, it is only in recent years that KDs have been considered as an adjuvant therapy for cancer. As in fasting, KDs shift the body’s fuel source from carbohydrate to fat, reducing blood glucose and insulin while increasing ketone bodies, the byproducts of fatty acid metabolism. These low-carbohydrate, high-fat diets present a metabolic disadvantage to cancer cells due to their dependence on aerobic glycolysis for fuel acquisition and, unlike normal cells, most types of cancer cells are unable to metabolize ketones for energy [1,2]. Accordingly, KDs exploit the Warburg effect to “starve” cancer cells of the glucose and insulin required for proliferation.

Despite the growing evidence of KDs’ possible anti-tumor benefits, reluctance still exists from healthcare providers to prescribe this dietary regimen for cancer patients. Concerns relate to the perceived restrictive nature of KDs and their potential to detract from quality of life in a patient population already facing considerable physical and emotional stress. However, there is almost no research evidence evaluating these concerns. Indeed, the literature examining KDs in cancer patients is sparse, and even fewer studies have included mental or physical functioning in their research outcomes. A recent systematic review [3] of studies examining KD in adult patients with cancer found only three previous studies that have assessed quality of life outcomes, highlighting the need for additional, larger investigations in this field. 

Although there are several potential alterations to physical and mental functioning during cancer and its treatment, perhaps the most common is cancer-related fatigue (CRF). The estimated prevalence of CRF ranges from 25% to 99% in patients undergoing treatment, and up to 30% of patients continue to report feeling fatigued years after successful treatment of their disease [4,5,6,7]. This phenomenon may extend to gynecological cancers; a small, qualitative study of symptom burden in ovarian cancer patients reported that 93% of the women experienced fatigue as a symptom prior to and during cancer treatments [8]. In addition, a considerable proportion of cancer patients report CRF to be moderate to severe in intensity, ranging from 29% among cancer survivors up to 45% of patients undergoing active treatment [7]. Interventions for CRF have included psychosocial strategies, exercise, yoga, acupuncture, massage therapy, as well as pharmacologic options, including psychostimulants and antidepressants [9,10]. However, despite the prevalence, severity, and potential duration of CRF, almost no research has focused on dietary interventions as a possible means to ameliorate fatigue. 

Although the evidence available is limited, previous research suggests that KDs may improve physical and mental well-being. A small trial among advanced cancer patients found that insomnia and emotional functioning improved over the course of a three-month KD intervention, and case reports also reflect enhanced cognitive function [11,12,13]. In regard to fatigue, a low-glycemic-load (though not ketogenic) diet has been associated with significantly lower reported fatigue when compared to a high-glycemic load diet in overweight and obese adults [14]. Previous research also indicates that low-carbohydrate diets do not increase hunger, as many presume, but rather reduce feelings of hunger as well as sweet and starchy food cravings [15,16,17]. 

The purpose of the present analysis was to evaluate and compare the effects of a KD and the lower-fat, American Cancer Society diet (ACS) on physical and mental health status, hunger and satiety, and food cravings in women with ovarian or endometrial cancer. Questionnaire data were obtained from a parallel-arm, randomized, controlled trial; results related to body composition and glucose metabolism from this trial have been previously reported [18]. We tested the hypothesis that, relative to the ACS, the KD would improve mental and physical function, including energy levels; reduce hunger; and diminish sweet and starchy food cravings in this patient population.

## 2. Methods

### 2.1. Participants

This trial was registered at ClinicalTrials.gov as NCT03171506. Details of the study have been published elsewhere [18]. Briefly, women diagnosed with ovarian or endometrial cancer were recruited from the University of Alabama at Birmingham (UAB) Gynecologic Oncology clinic and surrounding treatment centers between October 2015 and April 2017. Eligibility criteria were BMI ≥ 18.5 kg/m^2^, age ≥ 19 years, no medical condition affecting body weight (other than cancer and its associated treatment), and not attempting diet modification. Women with type 2 diabetes were eligible to participate with additional medical supervision from the study physician. UAB’s Institutional Review Board approved the study, and all participants provided written informed consent prior to enrollment.

### 2.2. Protocol

All participants completed two testing visits: one at baseline and one after 12 weeks of the assigned diet intervention (described below). After an overnight fast (≥10 h), participants reported to UAB’s Clinical Research Unit for a series of blood draws to measure a variety of biomarkers, including serum β-hydroxybutyrate (BHB), and to complete a series of questionnaires. Changes in body composition were assessed by dual energy X-ray absorptiometry (DXA) for all participants.

The questionnaires completed at each visit included:

Medical Outcomes Study Short Form-12 Health Survey (SF-12)

The Medical Outcomes Study Short Form-12 Health Survey (SF-12) [19,20] was used to measure physical and mental health status. Responses to the questionnaire’s 12 items are scored to produce a physical component summary and a mental component summary on a scale from 0 to 100, with higher scores indicative of better functional status. The SF-12 is well validated and has been used in over 600 clinical trials in people with a variety of illnesses and health conditions, including cancer [21,22].

Visual Analog Scale for Appetite (VAS)

Perceived hunger and satiety at the time of testing were assessed by self-report using a visual analog scale (VAS) for appetite [23,24]. The VAS consists of eight questions regarding hunger, fullness, and desire to eat sweet, savory, salty, or fatty foods. Participants indicate their response with an “X” on a 100 mm line anchored at either end by opposing responses (e.g., “I am completely empty”/“I cannot eat another bite”).

Food Craving Inventory (FCI)

The Food Cravings Inventory (FCI) [25] was used to assess food cravings in relation to four categories: high-fat foods, sweets, starches, and “fast food fats” (e.g., pizza, hamburgers, and potato chips). The FCI lists 24 food items, and respondents are asked to rate the extent to which they crave each of the foods, using a scale that ranges from 1 = *Never* to 5 = *Always*/*Almost Every Day*. The scoring of the questionnaire yields craving scores for each category as well as an overall craving score. 

### 2.3. Diet Interventions

This study was conducted as a randomized clinical trial with parallel-arm design. Participants were randomly assigned, using a computer-generated blocked randomization scheme (created by KRF), to one of two diet interventions: either the American Cancer Society diet (ACS) or the ketogenic diet (KD). A summary of each diet is listed in Table 1. The ACS diet was based on recommendations from the American Cancer Society and the Academy of Nutrition and Dietetics [26,27]. Participants assigned to this diet were encouraged to increase intake of fiber-rich fruits, vegetables, whole grains, as well as lean meats and small amounts of healthful fats. The KD consisted of approximately 5% of energy from carbohydrate (≤20 g/day), 25% from protein (≤100 g/day) and 70% from fat (≥125 g/day). Neither group was instructed to alter total energy intake. 

Diet-specific nutritional counseling was provided by a registered dietitian in individual face-to-face meetings immediately following the baseline testing visit. Participants received weekly phone calls and/or e-mails from the study dietitian for the remainder of the intervention to review food records and discuss strategies to enhance participants’ adherence and enjoyment of their assigned diets. Adherence for KD participants was also monitored using urinary ketone strips (Bayer AG, Leverkusen, Germany). 

### 2.4. Statistical Methods

Descriptive statistics were calculated for all study variables. All statistical tests were two-sided, with an alpha level of 0.05 denoting statistical significance. No adjustment was applied to the level of significance for multiple testing. Statistical analyses were performed using SAS (version 9.4, SAS Institute; Cary, NC, USA). Analysis of covariance (ANCOVA) was used to assess between-group differences and to test the hypothesis that the KD (1) improves mental and physical health, including energy levels, (2) reduces hunger, and (3) diminishes sweet and starchy food cravings relative to the ACS. Baseline values, chemotherapy status, and/or change in fat mass were used as covariates where appropriate. To assess differences in perceived energy between diet groups, sub-group analyses were conducted on the basis of chemotherapy status (i.e., current chemotherapy vs. no chemotherapy) due to the prevalence of chemotherapy-related fatigue [10]. Paired *t*-tests were used to assess within-group differences and to test the hypothesis that the KD (1) improves mental and physical health, including energy levels, (2) reduces hunger, and (3) diminishes sweet and starchy food cravings at 12 weeks when compared to the baseline. Pearson correlation analyses were used to estimate associations between questionnaire results and serum β-hydroxybutyrate concentration, a biomarker of circulating ketones. The version of the VAS was printed in a reduced format such that the line provided was 40 mm long rather than the standard 100 mm; all responses were multiplied by 2.5 to be converted to the 100-mm scale. The statistical analysis plan included an investigation for outliers, and the final analyses excluded them in order to lessen the influence of extreme observations on the overall results. One outlier (>3 standard deviations above the mean) was excluded from food craving analyses involving starches and high-fat foods. In addition, two participants in the KD group only partially completed the SF-12 at the follow-up visit; two participants in the KD group also did not complete all items on the VAS. Accordingly, these participants’ responses have been excluded from the corresponding analyses. 

## 3. Results

### 3.1. Patient Demographics

A total of 182 prospective candidates were screened for participation in this study, 73 women were randomized (ACS: *n* = 36; KD: *n* = 37), and 45 women (ACS: *n* = 20; KD: *n* = 25) completed their assigned 12-week diet intervention. After randomization, 16 women did not enroll due to scheduling conflicts, and 6 women in each diet group (*n* = 12) withdrew during the course of the study due to scheduling conflicts (*n* = 4), no longer wishing to comply with dietary requirements (*n* = 3), cancer recurrence (*n* = 3), and death (*n* = 2). Of the 45 participants who completed the study, the mean age of patients at baseline was 60.2 years (31–79 years), and the mean BMI was 31.7 kg/m^2^ (18.9–56.1 kg/m^2^). The study population was predominantly composed of Caucasian women (87%), with African American and Asian women making up 11% and 2% of the sample, respectively. More than half (62%) of the sample had been diagnosed with ovarian cancer, with the remaining 17 patients having been diagnosed with endometrial cancer. Of the 45 participants who completed the trial, 11 (24%) received concurrent chemotherapy while undergoing this diet intervention. There were no significant differences between diet groups (KD vs. ACS) in age, BMI, racial distribution, cancer type, proportion receiving concurrent chemotherapy, or time since initial cancer diagnosis. A total of 4 women with type 2 diabetes completed the study (ACS: *n* = 1; KD: *n* = 3). 

### 3.2. SF-12

**Figure 1:**
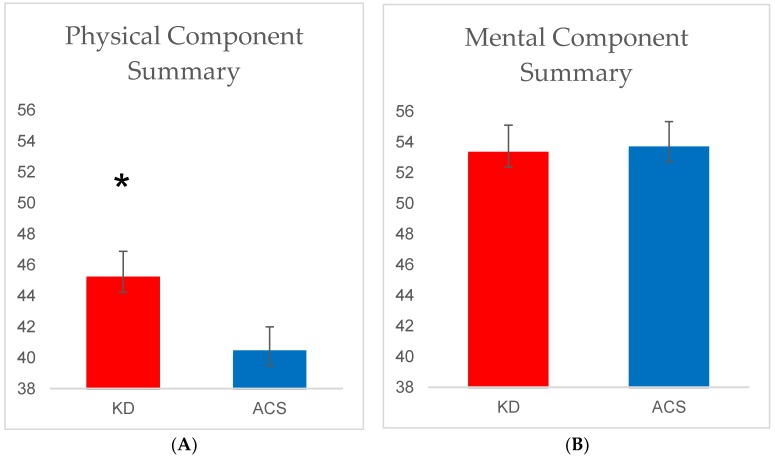
(**A**) Physical component summary scores at 12 weeks by diet group and (**B**) mental component summary at 12 weeks. Values are adjusted means ± SEM. * Denotes a significant between-group difference (*p* < 0.05) from ACS arm by ANCOVA, using baseline values and chemotherapy status as covariates. ACS, *n* = 20; KD, *n* = 23.

The between-group differences in physical component summary (PCS) and mental component summary (MCS) are shown in Figure 1. At 12 weeks, there was a significant between-group difference in the PCS after adjusting for baseline values and chemotherapy status (*p* = 0.04). When fat loss was added to the model, this effect was slightly attenuated (*p* = 0.064). In addition, there was a significant (11%) within-group increase in PCS from baseline to 12 weeks in the KD group (*p* = 0.02) but not in the ACS group (*p* = 0.67). There were no significant statistical differences in the MCS between or within the diet groups. Correlation analyses did not reveal any significant associations between the PCS or MCS and serum BHB concentration. 

The SF-12 question related to energy level (i.e., “How much of the time in the past four weeks did you have a lot of energy?”) was analyzed separately to assess whether there was a difference between diet groups; while not a direct measure, this item may reflect changes in fatigue. There was no significant difference in responses at 12 weeks between the diet groups. However, participants in the KD group who were not receiving concurrent chemotherapy reported a statistically significant (23%) improvement in energy level from baseline to 12 weeks (*p* = 0.02) while this was not the case among those in the ACS diet group not receiving concurrent chemotherapy (*p* = 0.11). Lastly, there was not a significant association between serum BHB and perceived energy. 

### 3.3. VAS for Appetite

Table 2 summarizes participant responses to the VAS by diet group. At 12 weeks, there was a significant between-group difference in the desire to eat something salty in that the KD group craved salt to a greater extent than the ACS group. This difference remained even after adjusting for baseline values and chemotherapy status (*p* = 0.03). There were no other significant between-group differences, and there were no significant within-group differences from baseline to Week 12. As previously reported, there was a significant between-group difference in serum BHB concentration at 12 weeks (group means: ACS, 0.25 ± 0.04 mmol/L vs. KD, 0.91 ± 0.16 mmol/L, *p* < 0.001), but correlation analyses did not reveal any significant associations between appetite, fullness, or desire to eat and serum BHB concentration.

### 3.4. FCI

Table 3 summarizes participant responses to the FCI by diet group. After 12 weeks of the diet intervention, there was a significant between-group difference in cravings for starchy foods and fast food fats after adjusting for baseline values and chemotherapy status (*p* = 0.03 and *p* = 0.04, respectively), with the KD group reporting less frequent cravings. There were no significant between-group differences in cravings for high-fat foods or sweets. Participants in the KD group reported significantly less frequent cravings for starchy foods (*p* = 0.003), sweets (*p* = 0.02), fast food fats (*p* = 0.004), and overall cravings (*p* = 0.0004) at 12 weeks when compared to baseline. There were no significant within-group differences for the ACS. Neither weight loss nor fat loss was associated with changes in food cravings.

## 4. Discussion

KDs result in several metabolic changes that may cause stress to cancer cells. However, clinical trials examining KDs’ anti-cancer effects are limited, and even fewer studies within this field have analyzed changes in quality of life, hunger, and food cravings. In this investigation, we tested the hypothesis that the KD would improve mental and physical function, reduce hunger, and diminish sweet and starchy food cravings in women with ovarian or endometrial cancer. Our results indicated that, compared to the ACS, the KD improved perceived physical functional status as well as reduced cravings for starchy food and fast food fats. Those in the KD group not receiving chemotherapy also reported significantly more energy at 12 weeks compared to baseline. These findings suggest that a KD is feasible for cancer patients, and may provide several benefits that improve quality of life. 

As noted, the PCS of the SF-12 was significantly higher in the KD than that of the ACS after the 12-week diet intervention. Previous studies have indicated that decreases in physical function coincide with increased mortality and morbidity in cancer survivors [28,29]. Moreover, the KD resulted in a clinically important improvement in physical status. Previous studies of patients with chronic conditions, including cancer, have estimated that a 2- to 5-point change in PCS may be considered clinically meaningful [30,31,32]; the mean difference in PCS for the KD group in this sample was approximately 4 points. Future investigations might examine whether such diet-induced improvements in physical function might reduce morbidity and mortality. Although the MCS did not differ significantly between the two diet groups, scores in both groups were comparable if not higher than those observed in other samples of cancer patients as well as healthy cancer-free adults [19,22,33,34]. Thus, an increase in MCS may have been difficult to achieve due to a ceiling effect. Nonetheless, it seems clear that neither dietary regimen (i.e., neither low-fat nor low-carbohydrate) negatively impacted mental well-being. 

In subgroup analyses, the KD also increased energy in women not receiving concurrent chemotherapy over the course of the 12-week intervention. An often mentioned benefit of KDs among adherents is heightened and stabilized energy levels [35]. It has been hypothesized that this energizing effect is related to elevated ketone bodies, although we did not find a significant association between serum BHB and reported energy level in this sample. The effect on energy levels was limited to women not receiving chemotherapy during the diet intervention, which may be due to the fact that women in active treatment may be in more advanced stages of the disease. Alternatively, it is possible that the energizing benefits of the KD were reduced in the context of chemotherapy, the side effects of which often include fatigue. As described above, research examining the potential relationship between diet and CRF is limited and not focused on macronutrient composition. Our findings suggest that carbohydrate restriction merits continued investigation to determine its potential role in mitigating CRF. 

There were no significant differences between or within the diet groups for perceived hunger, satisfaction, fullness, or prospective food consumption on the VAS. These results contradict findings from previous, larger investigations in overweight and obese adults, which indicate that low-carbohydrate diets result in reduced hunger when compared to low-fat diets [15,17]. A systematic review of the literature related to KDs also revealed that adherents reported less hunger and a reduced desire to eat, even in the context of energy restriction [16]. Discrepancies in our results from the aforementioned studies may relate to the timing of the administration of the questionnaires. All participants completed the VAS at a single time point during each visit, in a fasted state. Multiple administrations of the VAS (e.g., before and after a test meal) may have provided more insight into each diet’s effect on appetite. Additionally, participants were not provided specific calorie goals during the diet intervention, so it is possible that there was considerable variation in terms of whether participants were in positive or negative energy balance throughout the study, which may in turn have influenced feelings of hunger or satiety. Nonetheless, our findings suggest that a KD does not adversely affect hunger and appetite and thus is not any more restrictive than other conventional diets. 

Responses to the VAS and FCI also revealed that the KD group experienced significant changes in food cravings. First, the KD resulted in significantly higher cravings for salty foods compared to the ACS. The decrease in insulin levels and increase in ketone bodies that typically accompany KDs may cause a natriuretic effect [36,37,38]. Thus, this observation may be a manifestation of sodium depletion in the KD. After 12 weeks of the diet intervention, there were significant between-group differences in cravings for starchy foods and fast food fats, and within the KD group, there were significant reductions in cravings for starchy foods, sweets, fast food fats, and overall cravings at 12 weeks compared to baseline. These findings are similar to those of a two-year clinical trial in which obese adults were assigned to either a low-carbohydrate or low-fat diet. Those in the low-carbohydrate group experienced significantly greater decreases in cravings for starchy foods, sweets, and fast food fats in comparison to the low-fat group, whereas the low-fat group reported decreases in cravings for high-fat foods, suggesting that adherents of any given diet may crave restricted foods less frequently [17]. In the present study, it is clear that the KD generated less frequent cravings across several food categories, many of which offer limited nutritional value other than energy. Accordingly, it is possible that a KD alters food cravings such that adherents consume more nutrient-dense foods on a regular basis. More research is needed to determine how these changes in food cravings may affect outcomes in cancer patients. 

This study has several strengths. This investigation was part of the first randomized, controlled trial examining KDs in the context of cancer. In addition, to our knowledge this is the first study to examine KD’s effects on quality of life in women with ovarian or endometrial cancer. Our results are limited by the heterogeneous nature of the sample in that approximately 25% of the participants received concurrent chemotherapy while on their assigned diets. As stated previously, in all likelihood, the administration of the questionnaires only in a fasted state influenced the results reported here. Finally, although this trial is one of the largest conducted to date in this field, it is limited by a relatively small sample size, which was sufficient to detect only large between-group differences; larger studies are needed.

In conclusion, we found that, among women with ovarian or endometrial cancer, a KD does not diminish quality of life; indeed, it may improve physical function, increase energy, and diminish specific food cravings. These findings may generalize to other cancers associated with obesity, such as colorectal or post-menopausal breast cancers. However, further research is needed to determine for which cancer types and treatment regimens a KD may be most appropriate and to examine how a long-term KD may impact the lived experience of cancer patients. 

## Figures and Tables

**Table 1:** Dietary guidelines for patients.

KD	ACS
Avoid all grains, fruits, and starchy vegetables Eat at least 2 cups leafy green vegetables and 1 cup non-starchy vegetables daily Fat sources may include olive oil, coconut oil, avocados, butter, cream, mayonnaise, and small amounts of nuts Limit full-fat cheese to 4 ounces per day Permitted protein foods include (un-breaded) meat, poultry, fish, and eggs Emphasize foods rich in sodium, potassium, and magnesium	Incorporate fruits and vegetables into meals and snacks as often as possible Limit or avoid saturated fats from meats, cheeses, and margarines. Small amounts of fat from avocados, vegetable oils, and nuts are permitted. Eat an abundance of high-fiber foods, such as whole grains, beans, fruits, and vegetables Consume foods with added sugar in moderation

**Table 2:** Baseline and 12-week VAS responses by diet.

	ACS (*n* = 20)			KD (*n* = 23 ^a^)			
	WEEK 0	WEEK 12	Δ	WEEK 0	WEEK 12	Δ	*p*
Hunger (mm)	41.6 ± 26.9	46.1 ± 24.5	4.5 ± 30.8	37.0 ± 25.2	39.6 ± 31.8	2.6 ± 28.6	0.58
Satisfaction (mm)	23.4 ± 17.9	26.1 ± 23.4	2.8 ± 22.0	30.5 ± 27.7	35.0 ± 31.4	4.5 ± 26.8	0.61
Fullness (mm)	16.1 ± 22.0	21.8 ± 22.1	5.6 ± 22.1	26.8 ± 27.7	28.3 ± 32.3	1.4 ± 36.1	0.79
Prospective Food Consumption (mm)	64.1 ± 23.5	54.5 ± 19.0	−9.6 ± 24.7	54.2 ± 27.0	44.8 ± 23.8	−9.5 ± 31.4	0.27
Desire for something sweet ^b^ (mm)	60.3 ± 34.1	66.3 ± 28.9	6.0 ± 32.9	65.8 ± 31.2	71.6 ± 27.7	5.9 ± 40.0	0.64
Desire for something salty ^b^ (mm)	61.6 ± 26.6	65.5 ± 23.3	3.9 ± 28.1	60.5 ± 23.4	49.8 ± 23.7	−10.8 ± 24.6 *	**0.03**
Desire for something savory ^b^ (mm)	40.3 ± 28.7	42.0 ± 22.2	1.8 ± 33.5	49.1 ± 26.9	42.3 ± 23.1	−6.8 ± 25.4	0.73
Desire for something fatty ^b^ (mm)	69.4 ± 29.5	68.1 ± 30.2	−1.3 ± 30.2	61.3 ± 27.7	58.9 ± 20.0	−2.4 ± 30.2	0.39

Values are unadjusted means ± SD. *p* Values indicate between-group differences from ANCOVA, using baseline responses and chemotherapy status as covariates; bolded values are *p* < 0.05. Δ is difference between Week 12 and Week 0 responses such that negative values indicate a decrease.* indicates significant within-group difference from baseline to 12 weeks using paired *t*-test. ^a^ Missing values for two KD participants. ^b^ Questions structured such that larger numbers indicate less desire to eat the specific type of food.

**Table 3:** Frequency of cravings at baseline and 12-week by diet.

	ACS (*n* = 20)			KD (*n* = 25)			
	WEEK 0	WEEK 12	Δ	WEEK 0	WEEK 12	Δ	*p*
Starches ^a^	1.7 ± 0.7	1.7 ± 0.6	0.0 ± 0.8	1.8 ± 0.6	1.3 ± 0.6	−0.4 ± 0.6 *	**0.03**
High−fat foods ^a^	2.2 ± 0.5	2.2 ± 0.5	0.0 ± 0.4	2.2 ± 0.5	2.0 ± 0.5	−0.1 ± 0.4	0.33
Sweets	2.2 ± 0.6	1.8 ± 1.0	−0.4 ± 1.2	2.2 ± 0.7	1.9 ± 0.8	−0.3 ± 0.7 *	0.84
Fast food fats	2.1 ± 0.7	2.1 ± 0.6	0.0 ± 0.7	2.1 ± 0.4	1.7 ± 0.7	−0.4 ± 0.6 *	**0.04**
Overall cravings ^a^	2.0 ± 0.4	1.9 ± 0.5	−0.1 ± 0.6	2.0 ± 0.4	1.7 ± 0.5	−0.3 ± 0.4 *	0.11

Values are unadjusted means ± SD. *p* Values indicate between-group differences from ANCOVA, using baseline responses per food craving category and chemotherapy status as covariates; bolded values are *p* < 0.05. Δ is difference between Week 12 and Week 0 responses such that negative values indicate a decrease. * indicates a significant within-group difference from baseline to 12 weeks using a paired *t*-test. ^a^ One outlier in KD group excluded.

## Abbreviations

KD	ketogenic diet
ACS	American Cancer Society diet
CRF	cancer-related fatigue
SF-12	Medical Outcomes Study Short Form-12 Health Survey
VAS	Visual Analog Scale for appetite
FCI	Food Craving Inventory
BHB	β-hydroxybutyrate
